# Dr. George E. Goodfellow

**Published:** 1911-02

**Authors:** 


Dr. George E. Goodfellow, formerly of San Francisco, more recently of Los Angeles, died at the Angelus Hospital in the latter city December 7, 1910, aged 54 years. He graduated in medicine from the University of Wooster, Cleveland, in 1876, and soon became a prominent surgeon in California. He was for five years chief surgeon of the Southern Pacific railway lines in Mexico with headquarters at Guaymas, Sonora, and had been a surgeon during the war with Spain, seeing service in Cuba.

Dr. Goodfellow at the time of his death was a member of the American Association of Obstetricians and Gynecologists as well as of the American Medical Association.

